# Who is a community health worker? – a systematic review of definitions

**DOI:** 10.1080/16549716.2017.1272223

**Published:** 2017-01-27

**Authors:** Abimbola Olaniran, Helen Smith, Regine Unkels, Sarah Bar-Zeev, Nynke van den Broek

**Affiliations:** ^a^ Centre for Maternal and Newborn Health, Liverpool School of Tropical Medicine, Liverpool, UK

**Keywords:** Lay health worker, health workforce, role, scope of practice

## Abstract

**Background:** Community health workers (CHWs) can play vital roles in increasing coverage of basic health services. However, there is a need for a systematic categorisation of CHWs that will aid common understanding among policy makers, programme planners, and researchers.

**Objective:** To identify the common themes in the definitions and descriptions of CHWs that will aid delineation within this cadre and distinguish CHWs from other healthcare providers.

**Design:** A systematic review of peer-reviewed papers and grey literature.

**Results:** We identified 119 papers that provided definitions of CHWs in 25 countries across 7 regions. The review shows CHWs as paraprofessionals or lay individuals with an in-depth understanding of the community culture and language, have received standardised job-related training of a shorter duration than health professionals, and their primary goal is to provide culturally appropriate health services to the community. CHWs can be categorised into three groups by education and pre-service training. These are lay health workers (individuals with little or no formal education who undergo a few days to a few weeks of informal training), level 1 paraprofessionals (individuals with some form of secondary education and subsequent informal training), and level 2 paraprofessionals (individuals with some form of secondary education and subsequent formal training lasting a few months to more than a year). Lay health workers tend to provide basic health services as unpaid volunteers while level 1 paraprofessionals often receive an allowance and level 2 paraprofessionals tend to be salaried.

**Conclusions:** This review provides a categorisation of CHWs that may be useful for health policy formulation, programme planning, and research.

## Background

To achieve the health-related Sustainable Development Goals (SDGs) and universal health coverage (UHC) in the post-2015 period, adequate numbers of competent health workers are required to provide services in an enabling environment [1]. A decade ago, the World Health Organization (WHO) declared a crisis in the global health workforce, characterised by severe shortages, inappropriate skill mixes, and gaps in service coverage [2]. The magnitude of the health workforce shortage varies across contexts with rural regions and low- and middle-income countries being the worst affected, and with critical shortages greatest in sub-Saharan Africa [3].

Evidence suggests that the presence of community health workers (CHWs) can complement an overstretched health workforce and may be key to increasing the availability of, and access to, basic health services especially in hard-to-reach areas, thereby bridging the health equity gap [4,5]. However, the diversity of roles and inconsistent nomenclature of CHWs make it difficult for policy makers, programme planners, and researchers working with CHWs in different settings to have a common understanding of ‘who is a CHW?’ [6]. Furthermore, in contrast to professional health workers, there is remarkable diversity in the content and duration of CHWs’ training. Some CHWs undergo informal training, with varied training content and durations, taking place outside recognised training institutions. Other CHWs undergo formal training in nationally recognised training institutions with structured training content and duration [7,8].

There have been different attempts to define and categorise CHWs based on their roles, educational level, and remuneration [5,9]. However, there is a need to build on these approaches by using a methodical approach that accommodates the diversities in CHW definitions to identify the spectrum of CHW categories that will aid comparison with other groups of health workers. Health worker categories that are comparable across disciplines may be crucial to designing CHW roles and positions within multidisciplinary health teams [10].

We conducted a systematic review of the global literature in order to: (1) identify definitions of CHWs reported in the literature; (2) determine key themes in how definitions are reported; and (3) clarify use of the term CHW for policy makers.

## Methods

### Selection criteria

We included any type of paper that described CHWs, their roles, and ways of working; this included published peer-reviewed primary research as well as commentaries, editorials, and review papers. For primary research, we included studies from any discipline using any study design and methods. We also included grey literature (unpublished reports and evaluations) if it included descriptions of CHWs or explanations of their roles. We included literature that described CHWs working in any aspect of primary or community healthcare and any disease or health issue. Overall, we included published and unpublished papers reported in English. In contrast, we excluded papers not focused on CHWs or papers that focused on CHWs but lacked a definition or description of CHWs. Furthermore, we excluded papers that are not reported in the English language.

### Search strategy

We searched eight databases for peer-reviewed literature (CHW Central, CINAHL Plus, ERIC, Global Health, LILACS, MEDLINE, Popline, and Web of Science); two of these databases also contained grey literature (CHW Central and Popline). Keywords for the search included terms for CHW (e.g. ‘community health worker*’ and alternate terms for ‘CHWs’) and a term for definition (e.g. ‘defin*’). We identified a total of 66 alternate terms for CHWs through a preliminary literature search and database subject headings (see Supplemental File 1 for an example of the search conducted in MEDLINE). We excluded terms which can be classified as ‘health professional’ as defined by the WHO’s mapping of occupations [11]. We limited the database searches to literature published in English between January 2004 and March 2016. The first global report on the global health workforce crisis was published in 2004; this emphasised the inclusion of CHWs in country health plans and resulted in a renewed interest in CHWs and subsequently additional research and publications on the topic [12].

We also hand-searched the references of all identified papers to find further relevant literature containing definitions or descriptions of CHWs. When the full text was not available online (n = 2 papers), we contacted the primary authors by email to request a copy. Both primary authors provided electronic copies of the papers that were not available online.

#### Study selection

Titles and abstracts (or executive summaries) of 7,653 sources identified in the various databases were reviewed independently by two of the authors (AO and RU) to identify potentially relevant papers. The same authors obtained and independently reviewed the full text of 783 potentially relevant papers. When there was no consensus on inclusion or exclusion of a paper (n = 1), they sought the opinion of a third reviewer (NvdB).

#### Data synthesis

We used narrative synthesis [13] to summarise definitions and other information contained in the included papers in a structured way. We extracted information on the characteristics of the study and definitions of CHWs using standard tables which were then used to make comparisons across included papers. Using an inductive approach, we reviewed the definitions for reoccurring patterns of items that relate to our study objectives; these formed our codes (Supplemental File 3). We reviewed these codes for concepts that are related in meaning, thereby constituting sub-themes, and subsequently we grouped sub-themes with similar connotations into themes: (1) selection criteria, (2) roles and/or tasks, (3) training, and (4) remuneration. Finally, we compared the themes, sub-themes, and codes for congruency in meaning and developed narratives to describe the various themes and sub-themes.

## Results

### Description of included papers

**Figure 1.**
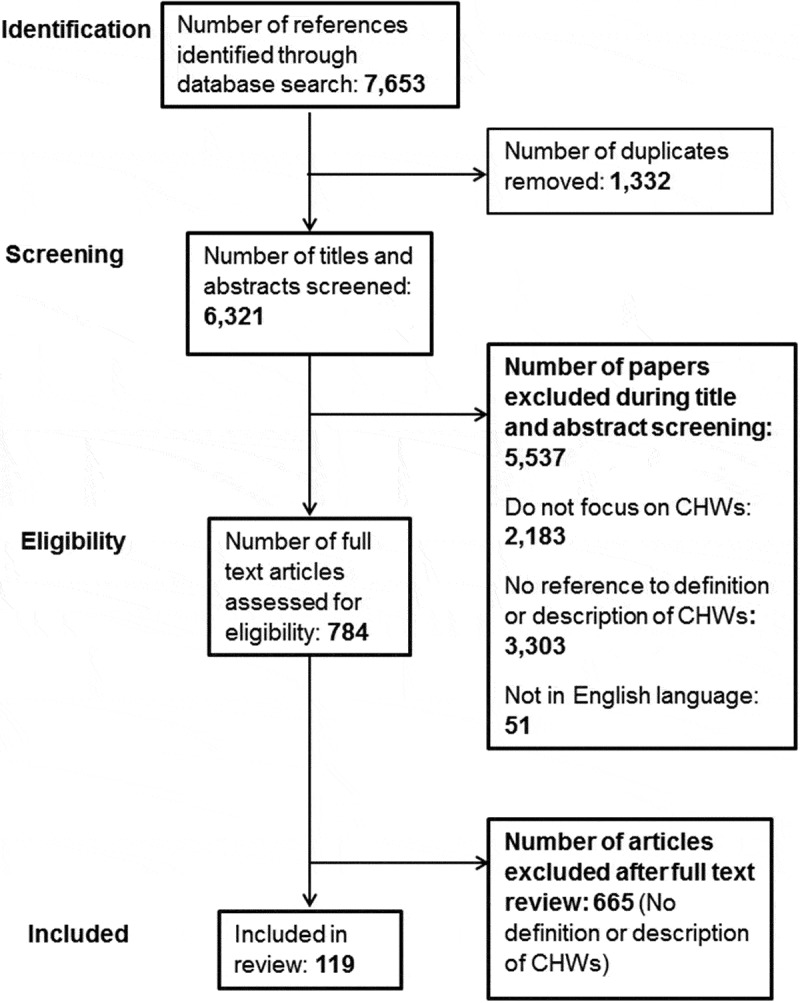
PRISMA flow diagram.

Figure 1 illustrates the process of study selection. The database searches identified 7,653 references; from these, we removed 1,332 duplicates. From the remaining 6,321 titles and abstracts, we excluded 5,537 as they were not in English or did not focus on CHWs. Of the 783 full text papers reviewed, we excluded 664 as they did not provide a definition or description of CHWs, leaving 119 included papers (Figure 1). Of the 119 included papers, 110 are peer-reviewed publications and 9 are grey reports or non-peer-reviewed papers. Table 1 summarises the characteristics of the 119 included papers; a full detailed list of the included papers and their characteristics is provided in Supplemental File 2.

**Table 1.** Key characteristics of included papers (n = 119).

Region		Income group		Year of publication		Type of publication	
East Asia & Pacific	3	High-income	62	2004–2009	19	Peer-reviewed	110
Europe & Central Asia	3	Upper middle-income	10	2010–2016	100	Grey literature	9
Latin America & the Caribbean	4	Low-income	16				
Middle East & North Africa	1	Multiple: across all income groups	16				
North America	59	Lower middle-income	15				
South Asia	13						
Sub-Saharan Africa	20						
Multiple countries	16

The majority (n = 100) of the papers documenting definitions of CHWs were published in the latter half of the review period (2010–2016) (Table 1). The included papers described CHWs in 25 countries across 7 regions and all income groups as defined by the World Bank [14]. Sixty-two (52%) of the included papers described CHWs in high-income countries, 10 (8%) in upper middle-income countries, 15 in (13%) lower middle-income countries, and 16 (13%) in low-income countries. Sixteen papers described CHWs in more than one country.

### Description of findings

Common themes identified in definitions or descriptions of CHWs are outlined in Supplemental Table 3. The common themes in definitions of CHWs include (1) selection criteria, (2) roles and/or tasks, (3) training, and (4) remuneration.

**Table 2.** Variation in the selection criteria used by stakeholders.

	Criteria used in selection of CHWs			
Organisation or institution involved in selection	Reside in the community	Personality traits that engender trust and respect	Previous experience providing healthcare	Educational qualification
Community	[15–18,19,20,21]	[21]		
NGOs	[22]			
Health facility management			[23,24]	[24]
Community and government	[25]			[25]
Government	[26]			[26,27]

**Table 3.** Pattern of educational qualification, pre-service training, and remuneration of CHW categories.

	Remuneration			
Educational qualification and pre-service training	Unpaid	Either unpaid or receive allowance/incentives	Receive allowance/incentives	Salaried
Individuals with minimal or no previous education and a few days to a few weeks of job-related pre-service training outside a recognised training institution	[22,90,116]	[53,78,124]		
Individuals with some secondary education and subsequent pre-service training outside a recognised training institution lasting a few months to more than a year			[35,92]	
Individuals with some secondary education and subsequent pre-service training in a recognised training institution lasting a few months to more than a year				[27,46,120]

### Selection of CHWs

Overall, more than half (n = 82) of the included papers described CHWs in relation to how they are selected from the community. Papers generally reported the selection criteria and the organisations or institutions involved in CHWs’ selection. Overall, CHWs are selected based on community membership, knowledge of the community culture and languages spoken, personality traits that encourage trust and respect, gender, previous experience providing healthcare, and educational qualification (Table 2).

Many of the included papers noted that CHWs are selected because they are community residents, who may not be indigenes of the community [15,22,28–37], or they are indigenous members residing in the community [16–20,25,26,38–72]. CHWs are also expected to have a close understanding or share the ethnicity, language [23,39,41–44,50,59,61,73–85], socioeconomic status [6,39,53,76,78], and life experiences [6,39,53,76,78,84] of the community. It is anticipated that these characteristics will ensure that CHWs can better mobilise and increase community members’ acceptance of the health services provided [61,64]. Some papers specified that CHWs should be trusted and respected community members [41,59,61,63,65,75,84,86,87], while in some contexts, CHWs are expected to possess leadership qualities [21,57,88].

In addition to these attributes, CHWs are expected to have other characteristics to better suit them for the role. In general, CHWs providing health services to women and children tend to be female [15,16,18,21,27,37,39,43,52,89–92] and those providing services in a health facility may be required to have some form of healthcare experience [23,24]. Previous primary or secondary education is sometimes considered in the selection of CHWs [24,28,29,46].

There are various organisations or institutions involved in the selection of CHWs. The included papers suggest that CHWs are either selected by the community members and leaders [15–21,28], the relevant department in the Ministry of Health on behalf of the government [29,39,47], non-governmental organisations (NGOs) [22], or by the health facility management [23,24]. The selection criteria, however, tend to vary across these stakeholder groups (Table 2). Being a resident of the community and having personality traits that engender trust and respect are often considered by the community and NGOs when selecting CHWs [15–22,38]. The CHWs selected by the government (or in collaboration with the community) are often selected on the basis of residence in the community and having some form of secondary education [25–27,29,39,93]. Health facility management often select CHWs on the basis of their level of education and having previous work experience in providing healthcare [23,24].

### The roles and tasks of CHWs

More than three-quarters (n = 90) of the included papers described the roles and/or tasks of CHWs in the community or within the health facility (Supplemental File 3). The roles of CHWs include health promotion and disease prevention, treatment of basic medical conditions, and collection of health data.

In relation to health promotion and disease prevention, CHWs are involved in activities both within the community and linked to the health facilities they are connected to. In the community, CHWs provide services to promote a healthy lifestyle and prevent disease [17,19,26,28,33,38,40,43,45,56,57,66,67,70,71,73,79,89,90,93–104], mobilise and encourage community members to utilise available health services [15,18,95,103,104], and facilitate access to facility-based healthcare by helping community members understand where to access care when needed [6,15,17,25,26,36,40,43,47,52,56,59,61,63–65,70,72,82,91,104–108]. Acting as ‘Patient Navigators’ [23,24,40,77,80,109–119], CHWs interpret health information and provide logistical support to patients accessing healthcare within a complex healthcare system. They also communicate health messages to community members [6,16,19,33,49,56,60,62,70,73,74,82–86,88,102,104,114,117,119–123], help patients to cope better with clinical conditions by providing psychosocial support [24,41,43,47,49,58,60,73,82,86,96,106,107], and serve as community representatives, providing a link between the community and health system [47,59,74,81,82,84,89,95,96,98,120]. Their role as a community representative entails conveying policy-related health messages to the community members and, in turn, reporting on community health needs and priorities to the health facilities they are linked to.

Some CHWs have additional roles of providing treatment for basic clinical conditions and minor ailments such as malaria and diarrhoea [15,38,39,44,55,95,99,122]. Other CHWs provide basic obstetric case management but the CHWs providing this have completed post-secondary formal training in order to provide these services [28,30,32,52]. Treatment of basic clinical conditions and management of basic obstetric cases appear to be part of a CHW’s role in low- and middle-income countries only; we did not find any evidence of CHWs providing these services in the papers from high-income countries.

The included papers suggest that CHWs also have a role in helping to collect and report, via existing mechanisms, information on the health status of the community members [30,59,93,97].

### Educational qualification and pre-service training of CHWs

Less than 20% (n = 21) of the included papers documented the educational qualifications or pre-service training of CHWs in the definitions used (Supplemental File 2). We identified three main patterns in the educational qualification and pre-service training of CHWs:

- Individuals with little or no formal education who have undergone a few days to a few weeks of job-related pre-service training outside a recognised training institution (e.g. training provided in a health facility by NGOs) [4,19,21,43,50,53,91,99,123,124].
- Individuals with some form of secondary education and subsequent job-related pre-service training outside a recognised training institution lasting any time from a few days to a few weeks [35,92].
- Individuals with some form of secondary education and subsequent pre-service training in a recognised training institution lasting a few months to more than a year [18,26,27,32,39,46,52,68,125–127].

### Remuneration of CHWs

Some CHWs perform their tasks as unpaid volunteers [18,22,89,90,97,103,108,116,128]. Other CHWs are paid an allowance [19,21,49,94,99], performance-based incentives [35,92], or a formal salary [27,29,39,46,93,126] (Table 3). The form of remuneration is often influenced by the level of educational qualification and form of CHWs’ pre-service training (Table 3). CHWs with minimal or no education and subsequent informal pre-service training are likely to be unpaid or receive an allowance while CHWs with some form of secondary education with subsequent informal pre-service training are likely to receive some allowance or monetary incentive [35,92]. Conversely, CHWs with some secondary education and subsequent formal pre-service training are often salaried and paid by the government [27,46,126].

### CHWs’ service recipients

The service recipients of CHWs tend to vary with country income status (Supplemental File 3). In high-income countries, CHWs provide health services to ethnic minority and low-income populations within these countries [21,23,33,41,42,45,47–50,56–60,62,63,74–80,84–86,97,101,107,108,110,113,115,118–120,123]. The service recipients in these settings are usually individuals with non-communicable diseases such as cancers, cardiovascular diseases, chronic respiratory diseases, and diabetes [23,24,31,33,41,47–49,56,58,63,73,77,80,85,96,100,101,106,109,112,113,115–117,120]. Only one paper from a high-income country (United States) noted that CHWs provide health services related to communicable diseases [129] and four documented maternal and child health services to ethnic minority populations in the United States [42,43,88,114].

Conversely, in low- and middle-income countries, CHWs tend to provide services related to communicable diseases [19,22,28,35,44,55,98,99,128] and maternal and child health services [16–18,26–28,30,32,34,51,52,81,89–92,94,125,127,130,131]. Only two of the included papers from upper middle-income countries (Iran and South Africa) stated that CHWs provide services related to non-communicable diseases [46,121].

## Discussion

This review explores the various definitions and descriptions of ‘community health workers’ and identified the common themes in these definitions to understand the essential characteristics of health workers classified as CHWs. Our intention was to describe the various categories of CHWs to help clarify use of the term and reach a common understanding among key stakeholders in community health programme planning, policy, and research.

### Common themes in the definitions of CHWs

Our review shows that CHWs engage in health promotion and disease prevention including basic treatment and collecting community health information. Most of the included definitions of CHWs are based on roles and tasks of CHWs. Role-based classification has been used by other authors including a review of global literature which classified CHWs as specialists (have fewer health roles within a defined thematic area) and generalists (have more health roles across thematic areas) [5]. Other studies [132,133], however, show that many governmental organisations and NGOs continue to formally or informally add tasks to the job description of CHWs, thereby blurring distinctions between CHWs who are generalists or specialists. There are suggestions that the definitions and categories of health workers should be based on competency or educational qualification rather than on tasks or roles [10]. In general, categorisations of health workers tend to be competency-based as the level of competency usually informs the type of tasks assigned to any group of health workers and their position within the health system. This competency-based categorisation may be key to developing frameworks to aid common understanding among stakeholders irrespective of context and comparison of different groups of health workers. Furthermore, these categories may assist programme planners in designing job profiles for multidisciplinary health teams and assigning commensurate remuneration based on competency levels [134].

In line with our review findings, the level of competency and educational qualification of health workers will often determine roles/tasks, selection/recruitment, and remuneration [135].

Through this review, we found that the level of competency or qualifications used for categorising health workers varies across settings, as found in other research [136]. The International Labour Organisation (ILO) suggest that lay health workers may or may not possess basic literacy skills while paraprofessionals are individuals who have had some form of formal long-duration post-secondary (non-tertiary) education and professionals usually have some form of tertiary education [137]. We modified the ILO definition of paraprofessionals to accommodate the two levels of competency of CHWs who may be classified as paraprofessionals. These are level 2 paraprofessionals, who are individuals with some level of secondary education and subsequent formal training of longer duration in a recognised training institution; and level 1 paraprofessionals, who are individuals with some level of secondary education who subsequently received informal, short-duration, pre-service training. The third and lowest level of competency among CHWs includes lay health workers with little or no formal education but who have received informal job-related training.

**Figure 2.**
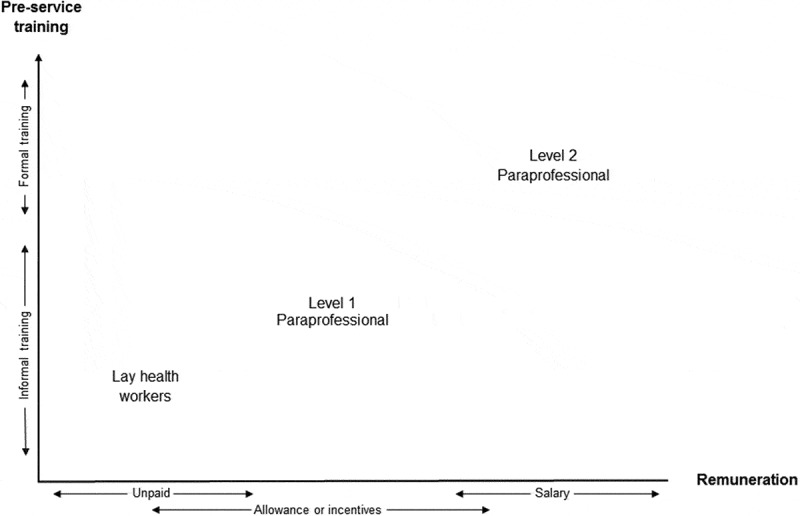
Linking pre-service training and remuneration to different levels of CHWs.

In addition, we noted that those CHWs who could be classified as lay health workers are usually unpaid volunteers or receive an allowance while those that match the definition of level 1 paraprofessionals often receive an allowance or incentive and the level 2 paraprofessionals are usually salaried. Figure 2 illustrates how the characteristics of educational qualification, pre-service training, and remuneration of the different levels are linked with the different categories of CHWs. It shows that the likelihood of having a salary increases with higher educational qualifications and longer duration of formal pre-service training.

In line with our review findings, the selection criteria and expected competencies of health workers often vary with the organisations/institutions involved in the selection [138]. These selection criteria will be crucial in identifying fit-for-purpose CHWs, especially as key stakeholders in global health continue to emphasise the advantages of a skills mix in health teams delivering primary care in areas with a health workforce shortage [1]. A clear understanding of the various levels of competencies of CHWs will guide programme planners in identifying and assigning roles to CHWs within the health system. Furthermore, a clear understanding of their competency levels and roles can inform policy and planning of CHW remuneration and career development [139].

### CHWs’ service recipients

Evidence shows that CHWs can increase health service coverage to hard-to-reach populations irrespective of country income status [140]. Our review noted that CHWs providing services in high-income settings mostly delivered services related to non-communicable diseases and mainly to hard-to-reach populations. Conversely, CHWs in low- and middle-income countries focus extensively on communicable diseases and maternal and child health and have relatively insignificant roles in the prevention and control of non-communicable diseases. There are indications that with appropriate health system support, CHWs can also contribute to the prevention and control of non-communicable disease [141] in low- and middle-income settings where about three-quarters of non-communicable disease-related deaths occur [142].

### Similarities and differences with definitions of similar cadres of health workers

The absence of distinct definitions for groups of health workers impairs planning and policy formulation for multidisciplinary health teams [143]. If we compare the common themes in definitions of CHWs with definitions of similar cadres of health workers, it is possible to determine the boundaries between each and make distinctions. Other relevant cadres of health workers include mid-level health workers [144] and Traditional Birth Attendants (TBAs) [145]. Hence, distinct CHW definition and categories may be key to assigning roles and positions to CHWs in multidisciplinary health teams.

Mid-level health workers are defined as frontline health workers with up to 3 years’ post-secondary school training to perform specific health-related tasks such as clinical or diagnostic functions, which are otherwise conducted by health professionals with a higher educational qualification [146,147]. This is similar to our findings which refer to CHWs as frontline health workers providing information and performing health-related tasks. The main difference is that CHWs receive training of fewer than 3 years. Additionally, our review shows that the primary goal of the CHW is to provide culturally appropriate health services to the community members.

TBAs have been described as individuals who assist mothers during childbirth and have acquired skills by conducting deliveries of babies on their own or through apprenticeship training received from other TBAs [145]. This definition of TBAs contrasts with our findings which suggest that CHWs have received a standardised job-related training in the context of the role they are expected to perform. However, trained TBAs who have received standardised job-related training in the context of an intervention could theoretically be considered as lay health workers but not paraprofessionals as they usually lack secondary education.

### Strengths and limitations

Due to resource and time limitations, we only included papers published in English and missed opportunities to review definitions of CHWs included in papers published in other languages (e.g. studies from francophone West Africa or Latin America). We included definitions of CHWs published from 2004–2016, excluding those pre-dating this period such as the definition proposed by the WHO Study Group in 1989:

Community health workers should be members of the communities where they work, should be selected by the communities, should be answerable to the communities for their activities, should be supported by the health system but not necessarily a part of its organization, and have shorter training than professional workers. [148]

We anticipate that we may have missed some definitions that still have contemporary relevance. However, we tried to make up for this by comparing common themes in the definitions included with those within definitions of CHWs pre-dating 2004 and found no diverging or alternative themes.

We acknowledge that our limited literature review to identify and include all alternative terms of CHWs may have missed some relevant terms. Overall, we used 66 alternative terms in this review and they were drawn from different contexts spanning the various socio-economic status and geographical regions; we, therefore, consider that this should be adequate to draw the needed inferences.

We did not assess the quality of papers included in this review because most were not primary research. None of the definitions contained in the included studies were based on any systematic or conceptual framework. To the best of our knowledge, our review is the first to use a methodical approach in defining ‘who is a CHW?’

## Conclusions

This review provides a methodical definition of CHWs based on common themes in CHW definitions, thereby clarifying use of the term for health policy makers, programme planners, and researchers. We acknowledge that a single definition may not project the diversity of the group nor is a universal definition desirable given that the concept of CHWs is highly political and has evolved to suit specific contexts, norms, and cultures. However, our review shows a link between pre-service training and remuneration and different levels of CHWs and this is helpful in distinguishing between CHWs as lay health workers or as paraprofessionals. In order to differentiate CHWs from other similar cadres of health workers, definitions of CHWs should emphasise that they are individuals with an in-depth understanding of the community culture and language, have received standardised job-related training which is of shorter duration than health professionals, and their primary goal is to provide culturally appropriate health services to the community.

### Recommendations for research and policy

There is a need for further research on sustainable fit-for-purpose models of remunerating lay health workers who are largely unpaid or receive short-term allowances from NGOs. These models may draw on existing models of remunerating levels 1 and 2 paraprofessional CHWs without compromising lay health workers’ accountability and commitment to the community. We, therefore, recommend that the models should reward high performance without compromising intrinsic motivating factors, teamwork, and commitment to the community. Fit-for-purpose and sustainable remuneration models may be key to long-term job satisfaction, commitment, and retention of CHWs, especially considering the resources invested in selecting, training, and equipping them before they start providing services.

## Supplementary Material

GHA_33313_Olaniran_suppl3.docx

GHA_33313_Olaniran_suppl2.docx

GHA_33313_Olaniran_suppl1.docx

## Paper context

Community health worker (CHW) definitions and categories are nuanced differently within and across contexts and stakeholder groups. Our review utilised a methodical approach in identifying the common themes in the various CHW definitions and subsequently developed a competency-based definition and categories that accommodate the peculiarities of the various contexts. This categorisation may inform a common understanding within and across stakeholder groups and also guide community health policy making, programme planning, and research across contexts.
