# Supplementary material for: Who is a community health worker? – a systematic review of definitions
Source: Glob Health Action. 2017 Jan 27;10(1):1272223. doi: 10.1080/16549716.2017.1272223 (PMC5328349; doi:10.1080/16549716.2017.1272223)
Supplement: GHA_33313_Olaniran_suppl3.docx [file zgha_a_1272223_sm9118.docx]

*Supplemental File 3*. An inductive approach to identifying the common themes in CHW definitions

| Theme | Sub-theme | Code | Description of CHW | Reference |
| --- | --- | --- | --- | --- |
| Selection criteria | Have an understanding of the community served | Community members | Indigenous members of the community served | (25-64) |
|  |  |  | Individuals who reside within the community | (15-24) |
|  |  | An understanding of the culture and language of the community served | Understand or share community culture, language or ethnicity | (16,26-29,36,42,48,51,65-78) |
|  |  | Similar socio-economic and life experiences to the community served | Share socio-economic status with community served | (6,42,68,70) |
|  |  |  | Share life experiences with community served | (6,42,68,70,76) |
|  | Job-related knowledge based on formal or informal education | Previous healthcare experience | Expected to have some healthcare experience | (77,88) |
|  |  | Some formal education | Expected to have some level of education | (18,25,32,37,39,41,59,88-90,119,120) |
|  | Specific skills and abilities that engender the trust and respect of community members | Trusted and respected individuals | Respected and trusted by community members | (26,48,51,53,55,67,76,79,80) |
|  |  |  | Possess leadership qualities | (46,81,82) |
|  | Gender consideration | Expected to be females | Female, preferably married in some cases | (17,24,28,30,39,41,82-87) |
| Roles and tasks of CHWs | Health promotion and disease prevention | Involved in health promotion | Provide services that promote the health of community members | (19,25,28,31,37,38,45,46,50,56,58,61,62,65,71,84,85,89-96) |
|  |  |  | Mobilise and encourage community members to utilise available health services | (17,39,89,97,98) |
|  |  |  | Facilitate access to facility-based healthcare by helping community members understand where to access care when needed | (6,17,23,25,28,33,37,38,41,45,48,51,53-55,61,63,64,74,86,98,110-113) |
|  |  | Patient navigation within the health facility | Patient navigation, helping patients to understand a complex healthcare system by translating the instructions and messages to them | (25,69,72,77,88,99-109) |
|  |  | Provide psychosocial support | Provide psychosocial support helping patients better cope with medical conditions | (26,28,33,35,47,49,65,74,79,88,90,111,112) |
|  |  | Health activist and advocates | Act as community representatives or advocates, serving as a link between the community and health system | (33,48,66,73,74,76,84,89,90,92,114) |
|  |  | Health education | Educate community members with health messages within the community | (6,19,30,35,39,45,49,50,52,61,65,66,74-79,78,79,81,96,98,104,114-116) |
|  |  |  | Health education within health facilities | (81,96,99,104,107,109,107) |
|  |  | Disease prevention | Provide health services to prevent diseases | (50,62,71,92,93,96-98) |
|  | Provide basic treatment | Provide basic case management and curative care | Provide basic case management of obstetric cases | (15,18,41) |
|  |  |  | Provide basic curative care for minor ailments | (17,29,44,89,93,117) |
|  | Health data collection | Collection of community health information | Collect information on the health status of community members and report to the formal health system | (15,48,91) |
| Educational qualification and pre-service training | Limited formal educational qualification and subsequent short duration informal pre-service training | Individuals with limited formal education but have undergone an informal job-related training lasting less than one month | Individuals with minimal or no previous, formal education but have undergone a job-related training. This pre-service training lasting between a few days to a month and does not take place in a recognised training institution | (4,28,36,42,50,82,86,117,118) |
|  |  | Individuals with limited formal education who have undergone informal job-related training lasting between one and six months | Individuals with minimal formal education and have undergone a job-related training lasting between one and six months which does not take place in a recognised training institution | (93) |
|  | Previous secondary education and subsequent short duration informal pre-service training | Individuals with some form of secondary education who have undergone an informal job-related training lasting less than six months | Individuals with some form of secondary education and have undergone a job-related training lasting a few weeks but less than six months. The job-related training is not in a recognised training institution | (119) |
|  | Previous secondary education and subsequent long duration formal pre-service training | Individuals with some form of secondary education who have undergone a formal job-related training lasting between six months and a year | Individuals with some form of secondary education and subsequent formal training in a recognised training institution lasting between six months and a year | (37,39,59,83,119) |
|  |  | Individuals with some form of secondary education who have undergone a formal job-related training lasting more than a year | Individuals with some form of secondary education and subsequent formal training in a recognised training institution lasting more than one year but less than three years | (18,32,41,120) |
| Remuneration | Unpaid | Unpaid | Unpaid volunteers | (21,39,84,85,91,97,106,113,121) |
|  | Unpaid or paid | May be unpaid or paid | Unpaid or receive an allowance or monetary incentives | (27,35,42,70,93,118) |
|  | Paid | Receive an allowance or incentive | Paid an allowance | (30,50,82) |
|  |  |  | Receive performance-based incentive | (22) |
|  |  | Receive a salary | Receive a formal salary | (32,92,120) |
| CHWs’ service recipients | Provide health services to individuals in high-income countries | Provide services to defined populations in high-income settings | Provide health services to ethnic minority and low-income populations in high-income settings | (19,26,27,31,33-36,45-49,52,53,66-72,76-79,82,90,95, 100,101,103,105,108,109,112-114,117) |
|  |  | Provide services to individuals who have (or at risk of) diseases in high-income countries | Provide services to individuals who have (or are at risk of) non-communicable diseases in high-income countries | (16,19,26,33-35,45,47,49,53,65,69,72,77,78,88,90,94, 95,99,102,103,105-107,111,114) |
|  |  |  | Provide services to individuals who have, or are at risk of, communicable diseases in high-income countries | (122) |
|  |  | Provide maternal and child health services in high-income countries | Provide maternal and child health services to ethnic minority populations in high-income countries | (27,28,81,104) |
|  | Provide health services to individuals in low- and middle-income countries | Provide services to individuals who have (or at risk of) diseases in low- and middle-income countries | Provide services to individuals who have, or are at risk of, non-communicable diseases in low- and middle-income countries | (32,115) |
|  |  |  | Provide services to individuals who have, or are at risk of, communicable diseases in low- and middle-income countries | (21,23,29,44,50,92,93,121) |
|  |  | Provide maternal and child health services in low- and middle-income countries | Provide maternal and child health services to an unspecified population in low- and middle-income countries | (15,17,18,20,30,37-41,73,83-87,119,123,124) |
