# Supplementary material for: Who is a community health worker? – a systematic review of definitions
Source: Glob Health Action. 2017 Jan 27;10(1):1272223. doi: 10.1080/16549716.2017.1272223 (PMC5328349; doi:10.1080/16549716.2017.1272223)
Supplement: GHA_33313_Olaniran_suppl2.docx [file zgha_a_1272223_sm9117.docx]

*Supplemental File 2*. Summary table of included papers

| **S/N** | **Author & year of publication** | **Paper type** | **Publication type** | **Country of service recipients** | **World Bank**  **income status*** | **Service recipients** | **Nomenclature** | **Definition** |
| --- | --- | --- | --- | --- | --- | --- | --- | --- |
| 1 | Keane D et al, 2004 | Grey literature | Commentary | USA | High-income country | Not documented | Community Health Worker (Promotoras) | Public health professionals who carry out a variety of health promotion, case management and service delivery activities at the community level. They come from the communities in which they work and act as advocates or representatives of those communities. They link individuals with needed health care by helping them understand and access an increasingly complicated healthcare system. |
| 2 | Anonymous, 2005 | Peer-reviewed | Descriptive study | India | Lower middle-income country | Not documented | Accredited Social Health Activist (ASHA) | ASHAs are female health activistc in the community who create awareness on health and its determinants and mobilise the community towards local health planning and increased utilization. They promote of good health practices and provide a minimum package of curative care as appropriate and feasible for that level. They have a formal education up to eight class. |
| 3 | Brownstein NJ et al, 2005 | Peer- reviewed | Narrative review | USA | High-income country | Patients with uncontrolled blood pressure, heart disease and stroke among an underserved population | Community Health Worker | Trusted, respected members of the community and their informal, but direct, involvement enhances the delivery of health related services. |
| 4 | Douthwaite M., 2005 | Peer-reviewed | Evaluation study | Pakistan | Lower middle-income country | Women | Lady health worker (LHW) | LHWs have a minimum of 8- year formal education and are resident of the community they serve. They undergo 15 months of training and receive a small allowance. Each LHW is attached to a government health facility. |
| 5 | Hinton A et al, 2005 | Peer-reviewed | Descriptive study | USA | High-income country | Cancer patients | Community Health Advisors | Usually, local women (and some men) who share cultural or ethnic characteristics with the population to be served. They often provide support and health education to community members in need of health improvement, along with promoting community capacity building. |
| 6 | Jandorf L et al, 2005 | Peer-reviewed | Evaluation study | USA | High-income country | Individuals at risk of colorectal cancer | Patient Navigator | An individual who works individually with patients to both educate and help them negotiate the medical system. |
| 7 | Lewin SA et al, 2005 | Peer- reviewed | Systematic review | International | Multiple countries | Not documented | Lay Health Worker | Any health worker carrying out functions related to healthcare delivery, trained in some way in the context of the intervention as well as having no formal professional or paraprofessional certiﬁcate or tertiary education degree. |
| 8 | Martin MY, 2005 | Peer-reviewed | Evaluation  study | USA | High-income country | Ethnic minority groups at risk of cancer | Community Health Advisor | Trusted helpers from within the community who provide emotional support, advice and facilitate tangible aid to members of their social network. Their understanding of community culture allows them to provide culturally appropriate, informal and spontaneous assistance to community members. |
| 9 | Health Resources and Services Administration (HRSA), 2007 | Grey literature | Technical report | USA | High-income country | Underserved populations | Community Health Worker | Lay members of the community who work either for pay or as volunteers in association with the local healthcare system in both urban and rural environments and usually share ethnicity, language, socioeconomic status and life experiences with the community members they serve. |
| 10 | Hill-Briggs F et al, 2007 | Peer-reviewed | Descriptive study | USA | High-income country | Urban African Americans with diabetes | Community Health Worker | Lay people who are trained to provide advocacy, support, counselling and information within the community. |
| 11 | Kennedy LA et al, 2008 | Peer- reviewed | Descriptive study | UK | High-income country | Food recipients in hard-to-reach neighbourhoods | Lay Food and Health Worker,  Lay Health Worker | They are paraprofessionals who are, usually, but not exclusively, recruited from outside the immediate social network and trained to fulﬁl slightly more specialist and demanding roles including duties usually undertaken by professionals (e.g. as health educators). Lay health workers should share the same social, cultural and ethnic backgrounds of the communities served. Any lay health worker: indigenous to the communities being served, carrying out functions related to community-based public health initiatives designed to prevent disease or promote health and wellbeing with a speciﬁc focus on food and public health; trained in some way in the context of the intervention, but having no formal professional or paraprofessional qualiﬁcations. |
| 12 | Petereit DG et al, 2008 | Peer-reviewed | Evaluation study | USA | High-income country | American Indian communities | Patient Navigator | Individuals who help their patients move through the complexities of the healthcare system. |
| 13 | Vargas RB et al, 2008 | Peer- reviewed | Descriptive study | USA | High-income country | Recipients of breast cancer care among African-American and Latino populations | Patient Navigator | Navigators are selected largely on the basis of being “dedicated people from the community” that are “sensitive to and can communicate with the population served”; subsequently they are taught or learn about the system that the patient will experience to “know the obstacle course, the terrain and the players”. Navigators are employees of the clinical site where they are based. |
| 14 | American Public Health Association (APHA), 2009 | Grey literature | Editorial | USA | High-income country | Not documented | Community Health Worker | Frontline public health workers who are trusted members of and/or have a deep understanding of the community served. This trusting relationship enables community health workers to serve as a liaison/link/intermediary between health/social services and the community to facilitate access to services and improve the quality and cultural competence of service delivery. |
| 15 | Bill DE et al, 2009 | Peer-reviewed | Descriptive study | USA | High-income country | Low-income pregnant Latina women | Promotoras | Bi-lingual and bi-cultural (Spanish speaking) indigenous women who participated in a comprehensive 115-hour training program to connect low-income pregnant immigrant Latina women with perinatal support and health promotion services. |
| 16 | Catalani CE et al, 2009 | Peer- reviewed | Descriptive study | USA | High-income country | Not documented | Community Health Worker | Trusted members of the community with unique access to and understanding of the community derived from shared ethnicity, culture, language and life experiences. This trust is vital to their work and descriptive of their practice. Public health professionals who work in a variety of environments and institutions on behalf of the community's health. |
| 17 | Darmstadt GL et al, 2009 | Peer- reviewed | Systematic review | International | Multiple countries | Newborns | Community-based Health Provider | Community-based Health Providers ideally live within the community in which they work, understand local culture and customs surrounding pregnancy and childbirth; and are likely to be well respected by community members; thus increasing the acceptability and uptake of interventions and galvanizing behaviour change. |
| 18 | De Jesus M et al, 2009 | Peer-reviewed | Descriptive study | USA | High-income country | Cape Verdean women | Health Promoter | Members of communities who work either for pay or as volunteers in association with the local healthcare system in both urban and rural environments. They usually share ethnicity, language, socioeconomic status and life experiences with the community members they serve. |
| 19 | Teti M et al, 2009 | Peer-reviewed | Descriptive study | USA | High-income country | People living with HIV/AIDS in a marginalised population | Peer Educator, Health Educator | Peers are often hired for their experience or commitment rather than their training; some may have little or no formal training. Peers differ from health educator because peers often share the culture, demographics and/or HIV serostatus of program recipients. |
| 20 | Granillo B et al, 2010 | Peer-reviewed | Descriptive study | USA | High-income country | Native Americans | Community Health Representative | A public health paraprofessional whose role as a community health educator and health advocate has expanded to become an integral part of the health delivery system of most tribes. They possess a unique set of skills and cultural awareness that make them an essential first responder on tribal land. As a result of their distinctive qualities, they have the capability of effectively mobilizing communities during times of crisis. They undergo a nationally accredited training program consisting of 48 hours of didactic sessions. |
| 21 | Herce ME et al, 2010 | Peer-reviewed | Evaluation study | Mexico | Upper middle-income country | Patients with tuberculosis | Health Promoters | Health promoters are mostly bilingual, speaking Spanish and one of the indigenous languages, conduct clinical and public health work in their communities. |
| 22 | Osawaa E et al, 2010 | Peer-reviewed | Evaluation study | Zimbabwe | Low-income country | People living with HIV/AIDS | Care Facilitator | Lay health worker working voluntarily for community. |
| 23 | Alvillar M et al, 2011 | Peer- reviewed | Evaluation study | USA | High-income country | Not documented | Community Health Worker | A frontline public health worker who is a trusted member of and/or has an unusually close understanding of the community served. This trusting relationship enables the community health worker to serve as a liaison/link/intermediary between health/social services and the community to facilitate access to services and improve the quality and cultural competence of service delivery. Builds individual and community capacity by increasing health knowledge and self-sufficiency through a range of activities such as outreach, community education, informal counselling, social support and advocacy. |
| 24 | Esparza A et al, 2011 | Peer-reviewed | Descriptive study | USA | High-income country | Vulnerable populations | Patient Navigator | Patient navigators seek to promote coordination of care by providing guidance for vulnerable individuals so that they may overcome barriers to timely, appropriate, high quality care. |
| 25 | Filgueiras AS et al, 2011 | Peer-reviewed | Evaluation study | Brazil | Upper middle-income country | Not documented | Community Health Agent | Community Health Agents act as links between families, users and the health service. |
| 26 | Herman AA et al, 2011 | Peer- reviewed | Commentary | USA | High-income country | Not documented | Community Health Worker | A trusted member of the community reﬂecting the linguistic and cultural diversity of the population served who plays an important role in connecting public and primary care to the communities that they serve. In some states, a more formal member of the integrated primary healthcare team and provides structured linkages between the community, the patient and the healthcare system. |
| 27 | Peacock N et al, 2011 | Peer-reviewed | Evaluation study | USA | High-income country | African American communities | Community Outreach Worker | Lay health workers who typically have close ties to the communities they serve and establish trusting relationships with clients and study participants. |
| 28 | Prata N et al, 2011 | Grey literature | Evaluation study | Ethiopia | Low-income country | Women | Community-based reproductive health agents (CBRHAs) | Lay health workers allowed to distribute oral contraceptives and condoms, for which they can receive a small commission. |
| 29 | Rosenthal EL et al, 2011 | Peer- reviewed | Evaluation studies | USA | High-income country | Not documented | Community Health Worker | Skilled community members who work with communities to improve health through a variety of strategies. |
| 30 | Shrestha BP et al, 2011 | Peer-reviewed | Evaluation study | Nepal | Low-income country | Children | Female Community Health Volunteer | A grassroots worker nominated by a mother’s group, responsible for building linkages with the health system at village level. They receive initial training on primary health care lasting 18 days. |
| 31 | Van Walleghem et al, 2011 | Peer-reviewed | Evaluation study | Canada | High-income country | Young adults with Type I diabetes | Patient Navigator | The patient navigator works closely with community-based health centers to promote community linkages and improve communication and not a health professional. |
| 32 | Agrawal PK et al, 2012 | Peer-reviewed | Evaluation study | India | Lower middle-income country | Newborns | Auxiliary Nurse Midwives (ANMs),  Angan Wadi Worker (AWW) | They are multipurpose CHWs employed by the government to promote various aspects of maternal and child health in India. ANMs provide counselling and health services to pregnant and postpartum mothers including birth attendance. AWWs are the community-based frontline workers selected from the community. They monitor and promote the growth of children. |
| 33 | Benskin LL et al, 2012 | Peer- reviewed | Narrative review | International | Multiple countries | Not documented | Village Health Worker | Lay person, rather than a health professional or a paraprofessional. A layperson who provides very basic, scientiﬁcally veriﬁed, curative care; educates and persuades within his or her own community to help implement illness-preventative and health promotion measures such as improved sanitation, hygiene and nutrition at locations where health professionals are not readily available. |
| 34 | Bonilla ZB et al, 2012 | Peer-reviewed | Descriptive study | USA | High-income country | Women | Promotoras | Promotoras are community leaders who are involved health education initiatives in both clinical and nonclinical settings. |
| 35 | Carver H et al, 2012 | Peer-reviewed | Evaluation study | UK | High-income country | Hard-to-reach, disadvantaged or underserved populations | Outreach Worker | Outreach workers work with members of an at-risk community in a variety of settings to improve access to health care and health improvement. A major task of outreach workers is to link clients with the services and resources they require, as well as providing support to improve coping, learning new skills and changing behaviour. Flexibility in these activities is viewed as essential. |
| 36 | de Heer HD et al, 2012 | Peer-reviewed | Evaluation study | USA | High-income country | Hispanic adults at risk of cardiovascular disease | Promotoras de Salud | Outreach health workers who are members of the community in which they work, serve as a liaison between healthcare providers and patients, providing various forms of health-related services, such as community advocacy, social support and cultural mediation. |
| 37 | Elkin EB et al, 2012 | Peer-reviewed | Evaluation study | USA | High-income country | Uninsured, low-income and high-risk populations | Patient Navigator | Lay health educators recruited from within the respective hospital systems or the surrounding communities, which are predominantly minority populations. They receive intensive initial training in a 1-week program orientation and subsequent ongoing training. |
| 38 | Farzadfar F et al, 2012 | Peer- reviewed | Evaluation study | Iran | Upper middle-income country | Patients with non-communicable diseases | Beharv | Community members with at least primary education. They undergo two years of classroom and practical training before beginning work in their local community and they receive a fixed salary. |
| 39 | George A et al, 2012 | Peer- reviewed | Evaluation study | Sub-Saharan Africa | Multiple countries | Children | Community Health Worker | Any health worker who carries out functions related to healthcare delivery is trained in some way to deliver an intervention and has no formal professional or tertiary education degree. |
| 40 | Gerber BS et al, 2012 | Peer- reviewed | Evaluation study | USA | High-income country | African-Americans and Latinos with diabetes. | Community Health Promoter | Health promoters provide education, evaluate medication use, promote behavioral change and self-management. They reinforce pharmacist and other provider recommendations. |
| 41 | Holt CL et al, 2012 | Peer-reviewed | Evaluation study | USA | High-income country | African American population | Community Health Advisor | Ethnically, linguistically, socioeconomically and experientially indigenous to the community, they serve as conduits of information, resources and services often to low-income populations. |
| 42 | Houston R et al, 2012 | Peer-reviewed | Narrative review | Nepal | Low-income country | Mothers and children | Female Community Health Volunteer | Female community health volunteers inform, educate and provide essential maternal and child health and family planning services at the community level. They are chosen by their community and reside in the communities. |
| 43 | Jacobson N et al, 2012 | Peer-reviewed | Descriptive study | Canada | High-income country | Individuals with mental health and/or addiction problems | Peer | Peers who work with clients as a coach, connector and partner to support and link clients to community-based supports and other resources. The peer role is explicitly a non-clinical one. |
| 44 | Jarvis L et al, 2012 | Grey literature | Technical report | International | Multiple countries | Not documented | Community Health Worker | CHWs are members of the communities where they work, who are selected by the communities, answerable to the communities for their activities, supported by the health system but not necessarily a part of its organization and have shorter training than professional workers. |
| 45 | Jean‐Pierre P et al, 2012 | Peer-reviewed | Evaluation study | USA | High-income country | Hispanic population with cancer | Patient Navigator | Patient navigators are trained to help patients effectively access and use healthcare resources in order to facilitate timely completion of recommended care and treatment. They vary in educational and socioeconomic backgrounds, ranging from lay health workers to healthcare professionals. |
| 46 | Larkey LK et al, 2012 | Peer-reviewed | Evaluation study | USA | High-income country | Underserved Latina Women at risk of cancer | Promotoras/es | Promotoras/es provide language-matched and culturally relevant health education, are networked in their community and have a deep understanding of how one might overcome logistical and psychosocial barriers to health behaviour change. |
| 47 | Naimoli JF et al, 2012 | Grey literature | Technical report | International | Multiple countries | Not documented | Community Health Worker | A health worker who receives standardised training outside the formal nursing or medical curricula to deliver a range of basic health, promotional, educational and mobilization services and has a defined role within the community system and larger health system. |
| 48 | Raj A et al, 2012 | Peer-reviewed | Evaluation study | USA | High-income country | Underserved patients diagnosed with breast cancer | Patient Navigator | Patient navigators facilitate access to quality medical care by identifying barriers to care and by bridging gaps in care through culturally sensitive coordination. They are trained lay workers who are culturally diverse and generally representative of the population served. |
| 49 | West Rasmus EK et al, 2012 | Peer- reviewed | Annotated bibliography | USA | High-income country | Underserved population | Promotoras/es de Salud | A cultural broker between their own community and the formal healthcare system and can play a crucial role in promoting health and wellness within their community. |
| 50 | Whop LJ et al, 2012 | Peer-reviewed | Literature review | USA | High-income country | Indigenous cancer patients | Patient Navigator | Community-based patient navigators are indigenous individuals who largely focus on awareness and prevention of cancer, networking and the maintenance of relationships with local health agencies; whereas, hospital-based patient navigators primarily assist indigenous people with cancer through their cancer-related treatment and social and emotional issues. The background of patient navigator ranges from lay individuals who are leaders in their community to registered nurses. |
| 51 | Zanchetta MS et al, 2012 | Peer- reviewed | Evaluation study | Brazil | Upper middle-income country | Shantytown individuals and families | Community Health Agent | Most community health agents are born, raised and live in the communities they serve and have formal education. They are to follow up with their clients, to ensure successful treatment and to protect, promote and restore their clients’ health. |
| 52 | Crigler L et al, 2013 | Grey literature | Guideline | International | Multiple countries | Not documented | Health Extension Worker | Usually paid, full-time employees but normally have about a year of initial training or less (in some cases, just a few weeks) and are generally recruited from the localities where they work. |
| 53 | Dynes M et al, 2013 | Peer-reviewed | Evaluation study | Ethiopia | Low-income country | Mothers and children | Health Extension Worker | Health extension workers are recruited from local villages, possess a 10th-grade education and are given one year of didactic and clinical health education. Health extension workers are expected to spend 75% of their time conducting community outreach with a focus on health promotion and serve as a connection between the home and health post. |
| 54 | Elkins T et al, 2013 | Peer-reviewed | Descriptive study | USA | High-income country | Underserved families in rural and inner city communities | Outreach Worker | Outreach workers are trained community mothers, who mentor their peers. They are residents of the target community, same race, culture and language use as the families served, provide services to underserved families in rural and inner city communities. They should complete at least 40 hours of initial training before they begin to serve families. |
| 55 | Frontline Health Worker Coalition (FHWC), 2013 | Grey literature | Descriptive study | International | Multiple countries | Not documented | Community Health Worker | Community health workers provide health education and referrals for a wide range of services and provide support and assistance to communities, families and individuals with preventive health measures and gaining access to appropriate curative health and social services. They create a bridge between providers of health, social and community services and communities that may have difficulty in accessing these services. |
| 56 | Gallo MF et al, 2013 | Peer- reviewed | Evaluation study | Madagascar | Low-income country | Recipients of injectable contraception | Volunteer Community Health Worker | Individuals who have received less training than professional healthcare providers and are typically members of the community they serve. |
| 57 | Gau Y et al, 2013 | Peer- reviewed | Evaluation study | China | Upper middle-income country | Not documented | Community Health Volunteer | The volunteers are individuals who are educated to assist residents by delivering health promotion and health monitoring activities. |
| 58 | Goff SL et al, 2013 | Peer- reviewed | Evaluation study | USA | High-income country | Low-income pregnant women | Patient Navigator | They assist patients in overcoming barriers to achieving healthcare goals. They assist patients attempting to access and interpret publicly reported information about the quality of care, tailoring the assistance to the patient’s needs. |
| 59 | Koskan A et al, 2013 | Peer-reviewed | Evaluation study | USA | High-income country | Hispanic population | Community Health Worker, Promotoras/es de Salud | Lay individuals trained to deliver health education and outreach to other members of their community. Promotoras/es de Salud function as integral members of healthcare teams, providing community-based outreach to marginalised populations and ideally, promotoras live, work and have existing social connections within the targeted community. |
| 60 | Koskan AM et al, 2013 | Peer- reviewed | Evaluation study | USA | High-income country | Hispanic population | Promotoras/es de Salud | Trusted and respected community members who engage in community outreach, participatory health education and provision of social support to others within their personal and community social networks. |
| 61 | Ramsey K et al, 2013 | Peer- reviewed | Commentary | Tanzania | Low-income country | Children under five years of age | Community Health Agent | Community health agents facilitate linkages between the community and the health system. A health worker who is formally trained and employed by the health system provides a package of health services in the community and connects people across the household to facility continuum. They are members of, selected by, and accountable to the communities where they work. |
| 62 | Raphael JL et al, 2013 | Peer- reviewed | Systematic review | International | Multiple countries | Children | Lay Health Worker | Lay health workers are individuals who perform functions related to healthcare delivery, have no formal or paraprofessional training, typically provided with informal job-related training. They may work in paid positions or as volunteers. |
| 63 | Singh P et al, 2013 | Peer-reviewed | Commentary | Sub-Saharan Africa | Multiple countries | Not documented | Community Health Worker | CHWs are volunteers who provide a few simple services, mostly in community awareness and disease prevention. |
| 64 | South J et al, 2013 | Peer- reviewed | Narrative review | United Kingdom | High-income country | Not documented | Lay Health Worker | Lay health worker share social status or common experiences and promote health and/or protect against different stressors. Their primary role is to act as a bridge between communities and health services, particularly where those communities experience health and social inequalities. Selected based on their knowledge of social networks and their ability to translate health messages to community members. Lay health workers are involved in mobilising community resources and building community capacity to address health issues. |
| 65 | Thom DH et al, 2013 | Peer-reviewed | Evaluation study | USA | High-income country | Low-income patients with diabetes | Community Health Worker, Peer Educator, Peer Coach | Community health workers are members of the same community as the patients they assist but do not necessarily have the same disease as the patient. Though some are volunteers, most are employed by a health facility or community agency. Peer educators and coaches, collectively known as peer supporters, in contrast, always have the same disease as the people they assist. Peer educators are usually volunteers who may receive a small monetary allowance and generally focus on providing ongoing support for self-management to a small group of clients. |
| 66 | Trejo G et al, 2013 | Peer-reviewed | Evaluation study | USA | High-income country | Latino farmworker families | Lay Health Promoter | Respected members of their community who understand community needs and share the culture, language and personal experiences of the people they serve. They use formal and informal teaching strategies to disseminate health information, increase awareness and empower people to change. |
| 67 | Wholey DA et al, 2013 | Peer- reviewed | Evaluation study | USA | High-income country | Patients with chronic health condition | Care Guide | Lay individuals who work with both providers and patients to achieve evidence-based chronic disease health goals such as keeping blood glucose readings and blood pressures within target levels. |
| 68 | Zulu JM et al, 2013 | Peer- reviewed | Evaluation study | Zambia | Lower middle- income country | Not documented | Community health worker (CHW) | Members of the communities where they  work, selected by their communities, and answerable to the communities for their activities. Although they may be supported by the health system as they perform a wide range of tasks that can be preventive, curative and  developmental in nature, they have less training than professional workers. |
| 69 | Ahmad MNS et al, 2014 | Peer-reviewed | Descriptive study | Afghanistan | Low-income country | Women | Community Health Worker | Most often community members who are trained, supported and supervised by more formal health professionals to deliver primary health services to their communities. |
| 70 | Allen JD et al, 2014 | Peer-reviewed | Evaluation study | USA | High-income country | Latinas (women) | Peer Health Advisor | Women from the church trained to deliver evidence-based screening interventions and reduction of structural barriers to screening. Peer Health Advisor candidates were selected by the pastor based on their leadership, communication and interpersonal skills. They complete two full days of training and receive a small stipend. |
| 71 | Balcazar HG et al, 2014 | Peer- reviewed | Evaluation study | USA | High-income country | Not restricted to any group | Community Health Worker, Promotoras/es de Salud | Community members serving as frontline public health workers, facilitating access to health and social services for those in need. CHWs serve as liaisons between health and social services and the community to facilitate access to services and service delivery, including health education and promotion services. |
| 72 | Barogui YT et al, 2014 | Peer-reviewed | Evaluation  study | Benin | Low-income country | Patients with Buruli ulcer | Community Health Volunteer | Lay individuals trained in a particular role of delivering curative or preventative care or control in their own community. |
| 73 | Brenner AT et al, 2014 | Peer-reviewed | Evaluation study | USA | High-income country | Vulnerable population | Patient Navigator | The patient navigator primarily facilitates completion of screening by addressing additional barriers that often supervene even after a screening test has been ordered. However, a patient navigator may also help to build on the knowledge, intent and self-efficacy established by decision aid viewing. |
| 74 | Condo J et al, 2014 | Peer-reviewed | Evaluation study | Rwanda | Low-income country | Mothers and children; individuals with communicable diseases | Community health worker (Binome and Animatrice de Santé Maternelle) | CHWs are required to have a minimum  of 6 years of education and are elected by their communities. Binomes are responsible for community health, nutrition and HIV/AIDS prevention. Animatrice de Santé Maternelle manage infant,pre and postnatal maternity care. |
| 75 | Das VNR et al, 2014 | Peer- reviewed | Evaluation | India | Lower middle-income country | Leishmaniasis patients | Accredited Social Health Activist (ASHA) | ASHAs are women who live in the community and receive performance-based incentives for overseeing maternal and other health-related issues in their village. |
| 76 | De La Cruz I et al, 2014 | Peer- reviewed | Evaluation study | USA | High-income country | Women with breast and cervical cancer screening abnormalities | Patient Navigator | Navigators are employees of the health centres trained to provide social support and address financial and logistical barriers to accessing cancer care. They have at least a high school education and some healthcare experience. |
| 77 | Gabitova G et al, 2014 | Peer-reviewed | Evaluation study | USA | High-income country | Breast cancer patient among ethnic minorities and low-income population | Patient Navigator | Navigators are bi-or multilingualism lay health workers with no clinical background who do not possess specific qualifications except for some prior experience in a hospital setting, strong communication skills and ability to work in a complex, multi-cultural setting with vulnerable patients. |
| 78 | Gobezayehu A et al, 2014 | Peer- reviewed | Evaluation study | Ethiopia | Low-income country | Mothers and children | Health Extension Worker,  Community Health Development Agent | Health extension workers are young women with ten years of primary and secondary school education and one year of certificate level health training. Community health development agents are members of the community who are chosen by the community or organization to work as volunteers in health-related activities such as community mobilization and health education. |
| 79 | Kaufmann LJ et al, 2014 | Peer- reviewed | Evaluation | USA | High-income country | American Indian and Alaska Native Veterans | Community Outreach Worker | A volunteer, a veteran and tribal community member who seeks out unenrolled native veteran, provides them with information on healthcare services and benefits and assist them with enrolment paperwork. The tribal veteran representative goes through extensive training every year. |
| 80 | Keating NL et al, 2014 | Peer- reviewed | Evaluation study | Mexico | Upper middle-income country | Women | Community Health Promoters | Individuals, almost all women, with little formal training and some basic health promotion training; they retain a non-salaried affiliation with local health clinics and undertake more basic, awareness-building activities. |
| 81 | Lopes SC et al, 2014 | Peer- reviewed | Evaluation studies | Guinea-Bissau | Low-income country | Children under five years of age | Community Health Worker | Members of the community who are recruited and trained in health prevention and promotion to provide services within their community. |
| 82 | Kelkar S et al, 2014 | Peer-reviewed | Descriptive study | India | Lower middle-income country | Not restricted to any group | Community Health Worker | Community members who work almost exclusively in community settings and serve as a link between healthcare consumers i.e. the community and healthcare providers. |
| 83 | Mangham-Jefferies L et al, 2014 | Peer- reviewed | Evaluation study | Ethiopia | Low-income country | Newborns | Health Extension Worker | Health extension workers are resident in the local community. They are females who are at least 18 years old and have completed the 10th grade of schooling. They receive one year of training and are paid a government salary. |
| 84 | Mash RJ et al, 2014 | Peer- reviewed | Evaluation study | South Africa | Upper middle-income country | People with Type 2 diabetes | Health Promoter | Health promoters are employed by community health centres who have been trained to deliver health education messages and to counsel patients. |
| 85 | Nandi S et al, 2014 | Peer-reviewed | Evaluation study | India | Lower middle-income country | Women who experience domestic violence | Mitanin | The Mitanins are women volunteers whose role is to undertake family level outreach services, community organization building and social mobilization on health and its determinants along with advocacy for the improvement of the health system. |
| 86 | Peu MD, 2014 | Peer- reviewed | Evaluation study | South Africa | Upper middle-income country | Families with adolescents orphaned by HIV and AIDS | Health Promoter | Health workers provide preventive and promotive health services. They are employed by the government and NGOs to act as advocates for various communities. |
| 87 | Pinto R et al, 2014 | Peer-reviewed | Descriptive study | Canada | High-income country | Non-specific community and South Asian community | Peer,  Lay Health Educator | Peers and lay health educators are members of the community but may not necessarily share a common health condition or concern. They live in the communities in which they work, understand what is meaningful to those communities, communicate in the language of the people and recognise and incorporate social buffers to help community members cope with stress and promote health outcomes. |
| 88 | Rasanathan K. et al, 2014 | Peer- reviewed | Evaluation study | Sub-Saharan Africa | Multiple countries | Children under 5 years of age | Community Health Worker | A health worker delivering health care in the community, trained in some way in the context of the intervention, having no formal professional or paraprofessional certificate or tertiary education degree; regardless of whether or not they receive payment. |
| 89 | Redick C et al, 2014 | Grey literature | Review | Sub-Saharan Africa and South Asia | Multiple countries | Not documented | Community Health Worker | Lay members of the community who are trained to provide basic health services. |
| 90 | Sarfraz M et al, 2014 | Peer-reviewed | Evaluation study | Pakistan | Lower middle-income country | Women in the reproductive age group | Lady Health Worker | A resident of the catchment area, aged between 18 and 45 years, preferably married and have had at least eight years of schooling and subsequently receive 15-month facility and community-based training. They act as a liaison between the formal health system and community, disseminate health education messages and provide health services. |
| 91 | Sarmento DR, 2014 | Peer- reviewed | Review | Timor-Leste | Lower middle-income country | Women | Community Health Worker | Lay members of the community who work exclusively to serve people who have lacked access to adequate care and establish vital links between healthcare consumers and providers to promote health in community settings. In general, they have been defined as the members of community who are selected by the communities to provide care for a broad range of health issues to the poorest and most vulnerable communities. |
| 92 | Tran NT et al, 2014 | Peer- reviewed | Systematic review | International | Multiple countries | Not restricted to any group | Community Health Worker | Lay people who live in the communities where they work and function as a critical link between these communities and the primary healthcare system. |
| 93 | Uriarte JA et al, 2014 | Peer-reviewed | Descriptive study | USA | High-income country | Not documented | Community Health Worker | Lay members of communities who are either paid or volunteer in association with the local healthcare system in both urban and rural environments and usually share ethnicity, language, socioeconomic status and life experiences with the community members they serve. They undergo a certification program requiring applicants to complete a 160-hour course. |
| 94 | Wayne N et al, 2014 | Peer-reviewed | Evaluation study | Canada | High-income country | People with diabetes from a modest socioeconomic strata community | Health Coach | Health coaches are individuals who primarily focus on helping patients define and attain personal goals and discover intrinsic health-oriented motivations. |
| 95 | Zulu JM et al. 2014 | Peer-reviewed | Evaluation study | Zambia | Lower middle-income country | Mothers and children; individuals with minor ailments | Community-based health worker (CBHW), Community health assistant (CHA) | CBHW are members of communities who work either for pay or as volunteers in association with the local health care system and usually share ethnicity, language, socio-economic status and life experiences with the community members they serve.  CHAs are recruited by the MoH, with the support of the community leaders and the Neighbourhood Health Committees (NHCs). They are registered by health professional bodies, receive a standardised 1-year training after secondary school education and they are on government payroll. |
| 96 | Abrahams-Gessel SM et al, 2015 | Peer-reviewed | Evaluation study | Bangladesh, Guatemala, Mexico and South Africa | Multiple countries | Patients at risk of cardiovascular disease risk | Community Health Worker | Health workers without traditional professional training, residing in the community and fluent in the community’s dominant language. |
| 97 | Charlot M et al, 2015 | Peer-reviewed | Evaluation study | USA | High-income country | Patients at risk of cancer | Patient Navigator | Patient navigators serve as patient advocates embedded within the clinical care practice and conduct their work with access to clinical providers as well as scheduling and administrative personnel. They work to reduce barriers to care by helping patients acquire health insurance and gain access to care; address logistical barriers such as scheduling, transportation and child care; and educate patients to improve knowledge, facilitate communication between patients and their providers and encourage patients to follow through with their care. |
| 98 | Cherrington AL et al, 2015 | Peer-reviewed | Evaluation study | USA | High-income country | African American patients with diabetes | Community Health Worker | CHWs are lay individuals who are actively part of their communities and either have diabetes themselves or have helped provide support to a close family member or friend with diabetes. |
| 99 | Cook JA et al, 2015 | Peer-reviewed | Evaluation study | USA | High-income country | Individuals with mental health challenges in low-income and at-risk population | Community Health Worker | Indigenous members of patients’ communities who have been trained to provide support, education and care coordination to improve medical outcomes for low-income and at-risk populations. |
| 100 | Dye CJ et al, 2015 | Peer-reviewed | Descriptive study | USA | High-income country | Hypertensive patients | Community Health Coach | CHWs are helpers who can be effective in facilitating education, behavior change, health self-management and access to health care among underserved and hard-to-reach populations recruited from the local community. |
| 101 | Farah FN et al, 2015 | Peer-reviewed | Evaluation study | India | Lower middle-income country | Mothers and children | Accredited Social Health Activist | A woman selected by the community, resident in the community and who is trained, deployed and supported to function in her own village to improve the health status of the people through securing their access to healthcare services. Her job responsibilities are three-fold, including the role of a link-worker (facilitating access to healthcare facilities and accompanying women and children), that of a community health worker (depot-holder for selected essential medicines and responsible for treatment of minor ailments) and of a health activist (creating health awareness and mobilizing the community for change in health status). |
| 102 | Fischer SM et al, 2015 | Peer-reviewed | Evaluation study | USA | High-income country | Latinos with serious illnesses | Patient Navigator | Often lay people who are part of or identify with the community with which they work. Their role is to educate, activate and advocate for patients and families, addressing barriers to care. |
| 103 | Give CS et al, 2015 | Peer-reviewed | Evaluation | Mozambique | Low-income country | Individuals with malaria, acute respiratory infection, diarrhoeal diseases, sexually transmitted infections and HIV infection | Agentes Polivalentes Elementares | Multi-purpose agents who have received a 4-month training reflecting the package of preventive, promotive and curative services. They are volunteers who sign an agreement, describing their right to an allowance and access to free health care at the local health centre. |
| 104 | Jimenez DE et al, 2015 | Peer-reviewed | Evaluation study | USA | High-income country | Older Latinos (Aged 60+) at risk of mental health challenges | Community Health Worker | Lay community members who work almost exclusively in community settings and effectively connect consumers to providers in order to promote health and prevent diseases among groups that have traditionally lacked access to adequate care. |
| 105 | Johnson SL et al, 2015 | Peer-reviewed | Descriptive study | USA | High-income country | Socially and economically disadvantaged groups | Community Health Worker | Trusted members of and/or have an unusually close understanding of the community they serve. They often focus on bridging cultural divides between patients, communities, healthcare providers and healthcare systems. They also engage in policy advocacy and community-based research aimed at improving conditions necessary for health. |
| 106 | Kowitt SD et al, 2015 | Peer- reviewed | Evaluation study | Thailand | Upper middle-income country | Individuals with communicable diseases, non-communicable diseases and maternal and child health | Village Health Volunteer | Community members selected from the community. They receive seven days of training in primary health care and 15 days of specialised on-the-job training in health promotion, disease prevention and health education. They are given a monthly THB600 (about USD20) government allowance to assist with implementing their duties. |
| 107 | Moshabela M et al, 2015 | Peer- reviewed | Evaluation study | South Africa | Upper middle-income country | Chronically ill patients with communicable diseases | Community Care Worker | They are recruited from within and around the local community by home-based care organisations and trained to provide basic services as volunteer caregivers to people in their home. |
| 108 | Mumtaz Z et al, 2015 | Peer-reviewed | Evaluation study | Pakistan | Lower middle-income country | Poor, marginalised and disadvantaged women | Community Midwife | They are trained to attend normal childbirths and to recognise and refer obstetric complications through the establishment of private practices in their home villages. Their training includes a 12-month classroom component followed by a 6-month practical clinical component. They are expected to meet education and residency criteria. |
| 109 | Nkwo PO et al, 2015 | Peer-reviewed | Evaluation study | Nigeria | Lower middle-income country | Not documented | Community Health Extension Worker,  Junior Community Health Extension Worker | The community health extension worker is trained for three years to provide basic public health services in primary healthcare clinics and in the communities. The junior community health extension worker is trained for 2½ years on the same skills as the community health extension worker and is expected to assist the community health extension worker in his/her duties. Both categories of community health extension workers are officially designated as community health extension worker although their starting salary levels are different. The community health extension worker and junior community extension worker have no competency-based midwifery training |
| 110 | Ramírez DM et al, 2015 | Peer-reviewed | Evaluation study | USA | High-income country | Small and home-based Hispanic businesses | Promotoras | A female, Hispanic community member who has leadership qualities allowing her to effectively promote a particular issue in her own community. She partners with organizations to assist them in achieving common goals and is indigenous to the community where she works. |
| 111 | Ranaghan CP et al, 2015 | Peer-reviewed | Systematic review protocol | USA | High-income country | Adult patients, 18 years and older in ambulatory care settings | Patient Navigator | A trained person who helps patients in overcoming barriers to care and use the healthcare system effectively and efficiently. They do not provide clinical care and may not be clinically oriented individuals. They can be non-professionals such as lay persons or volunteers. |
| 112 | Ruben K et al, 2015 | Peer-reviewed | Descriptive study | USA | High-income country | Low-income emergency department patients | Patient Navigator | They are to “provide culturally and linguistically competent health services”, help with patient access and interpretation/translation of languages. They are based in hospitals and community health centers throughout the country. |
| 113 | Sikander S et al, 2015 | Peer-reviewed | Evaluation study | India Pakistan | Lower middle-income countries | Mothers with perinatal depression | Peers | Women with children living in the same community as the clients, who are trained and supervised in delivering the intervention and work in partnership with established CHWs. |
| 114 | Singh D et al, 2015 | Peer-reviewed | Evaluation study | Uganda | Low-income country | Mothers and newborn in rural regions | Community Health Worker | Lay individuals, usually residents in the community in which they work who are trained in some aspect of health care. |
| 115 | Zulu JM et al, 2015 | Peer-reviewed | Evaluation study | Zambia | Lower middle-income country | Individuals with minor ailment | Community health assistant (CHA) | CHAs undergo a one-year standardised training programme and are registered with a regulatory body, perform much broader tasks, and on the government payroll. CHAs conduct health promotion activities, prevention as well as testing for and treating minor illnesses. They develop registers on the total number of people and common diseases in the community |
| 116 | Colleen B et al, 2016 | Peer-reviewed | Review | USA | High-income country | Not documented | Community Health Worker | A frontline public health worker who is a trusted member of and/or has an unusually close understanding of the community being served. This trusting relationship enables the CHW to serve as a liaison/link/intermediary between health/social services and the community to facilitate access to services and improve the quality and cultural competence of service delivery. |
| 117 | Johnston HB et al, 2016 | Peer-reviewed | Evaluation study | Ethiopia, India and South Africa | Multiple countries | Women seeking medical abortion | Health Extension Workers, Accredited Social Health Activist, Community-based Educators | Health extension workers have a minimum of 10 years of basic education and 12 months of public health training. Accredited social health activists have a minimum of 8 years of basic education and 20 days of public health training or village health workers with a minimum of 5 years of basic education and 28 days of public health education. Community-based educators have a minimum of 12 years of basic education and 20 days of public health training. |
| 118 | Sarin E et al, 2016 | Peer-reviewed | Descriptive study | India | Lower middle-income country | Mothers and children | Accredited Social Health Activist (ASHA) | ASHAs are female CHWs selected from the village to deliver health services and receive incentive for the activities. The ASHA is required to have completed eight years of education. |
| 119 | Silva R et al, 2016 | Peer- reviewed | Evaluation study | Ethiopia, Malawi, Mali | Low-income country | Mothers and children | Health Extension Worker (HEW), Health Surveillance Assistant (HSA) and Lay volunteer | HEWs are female CHWs who are paid government workers with an average of 10 years of formal schooling and resident in their catchment area. HSAs are male and female CHWs who are paid health workers with approximately 10 years of schooling, but sometimes not a resident in their catchment area. Lay volunteer are community volunteers who are resident in their local community and are nominated by it. |
