# Supplementary material for: Who is a community health worker? – a systematic review of definitions
Source: Glob Health Action. 2017 Jan 27;10(1):1272223. doi: 10.1080/16549716.2017.1272223 (PMC5328349; doi:10.1080/16549716.2017.1272223)
Supplement: GHA_33313_Olaniran_suppl1.docx [file zgha_a_1272223_sm9116.docx]

*Supplemental File 1*: Search strategy

We conducted a preliminary exploration of the literature for alternate terms of community health workers and searched databases for subject terms of community health workers. For the purpose of this review, we excluded terms which can be classified as health professional, as defined by the WHO’s mapping of occupations (11). In all database searches, we set search limits for date of publication (2004-2016) and language (English)

**MEDLINE search**

#1. Community health worker

#2. Health promoter

#3. Health educator

#4. (“Community health extension worker” OR “ Lady health worker” OR “Health coach" OR "Community health advisor" OR "Family advocate" OR "Outreach worker" OR "Peer counsellor" OR "Patient navigator" OR "Health interpreter" OR "Public health aide" OR "Community Health Agents" OR “Community Health Assistant” OR “Maternal Health Worker” OR “Community Nutrition Worker” OR “Maternal & Child Health Promotion Workers” OR “Community-based Worker” OR Community-based Health Worker” OR “Maternal Child Health Worker” OR “Nutrition Worker” OR OR “Mental Health Worker” OR “Postnatal Support Worker” OR “Community-based Skilled Birth Attendant”)

#5. (“Lay health worker” OR “Volunteer health worker” OR “Village health worker” OR Village Malaria Worker” OR “Female Community Health Volunteer” OR “Voluntary Malaria Worker” OR “Nutrition Volunteer” OR “Community Health Volunteer” OR “Village Health Guide” OR “Community Drug Distributor” OR “Village Health Helper” OR “Mother Coordinator” OR “Village Drug-Kit Manager” OR “Community Reproductive Health Worker” OR “Lay Health Visitor” “OR “Community Volunteer” OR “Community Health Advocate” OR “Community Health Aide” OR “Village Health Promoter” OR “Rural Health Worker” OR “Traditional Midwife” OR “Community Volunteer” OR “Lay Counselor” OR “Volunteer Counselor” OR “Volunteer Peer Counselor” OR “Peer Support Worker)

#6. ("Shasthyo Sebika" OR "Agente Comunitario de Salud" OR "Saksham Sahaya" OR "Visitadora" OR “Anganwadi Workers” OR “Promotoras de Salud” OR “Raedat” OR “Accompagnateur” OR “Behvarz” OR “Kader Posyandu” OR “Brigadistas” OR “Colaborador Voluntario” OR “Dai” OR “Bidan Kampong” OR “Dayas” OR “Doot”)

#7. (#1 OR #2 OR #3 OR #4 OR #5 OR #6)

#8. Defin*, this term only

#9. #7 AND #8
